# Visual Case Report of a Prosthetic Joint Infection and Chronic Dehiscence following a Total Knee Arthroplasty

**DOI:** 10.5070/M5.52251

**Published:** 2026-07-31

**Authors:** Kevin Chao, Alisa Wray, Danielle Matonis

**Affiliations:** * Anne Burnett Marion School of Medicine at TCU, Fort Worth, TX; ^ University of California, Irvine, Department of Emergency Medicine, Orange, CA

## Abstract

This case report highlights a unique presentation of a prosthetic joint infection. A 63-year-old female presented to the emergency department for right knee pain after falling three months earlier, shortly after a total knee arthroplasty. On exam, she was found to have a chronically dehisced wound with exposure of her femur, prosthetic, and overlying cellulitis. She was started on intravenous antibiotics and admitted to the orthopedic surgery service for an above-the-knee amputation. Her unusually delayed presentation and repeated refusal of operative management were heavily influenced by social determinants of health, including polysubstance use, lack of caregiver support, and housing instability.” This uncommon presentation of chronic dehiscence and prosthetic joint infection provides an opportunity to review the management of prosthetic joint infections compared to native joint septic arthritis, how socioeconomic factors drive patient outcomes, and a discussion on medical capacity.

**Topics:**

Wound dehiscence, chronic dehiscence, prosthetic joint infection, septic joint, total knee arthroplasty, social determinants of health, medical capacity.

**Figure:**
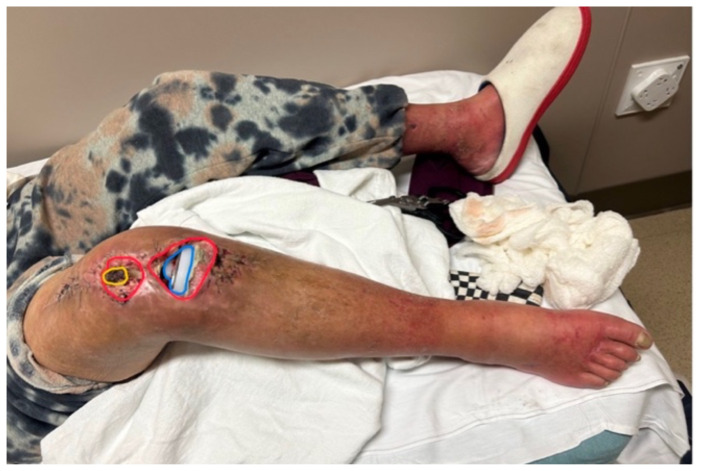


**Figure:**
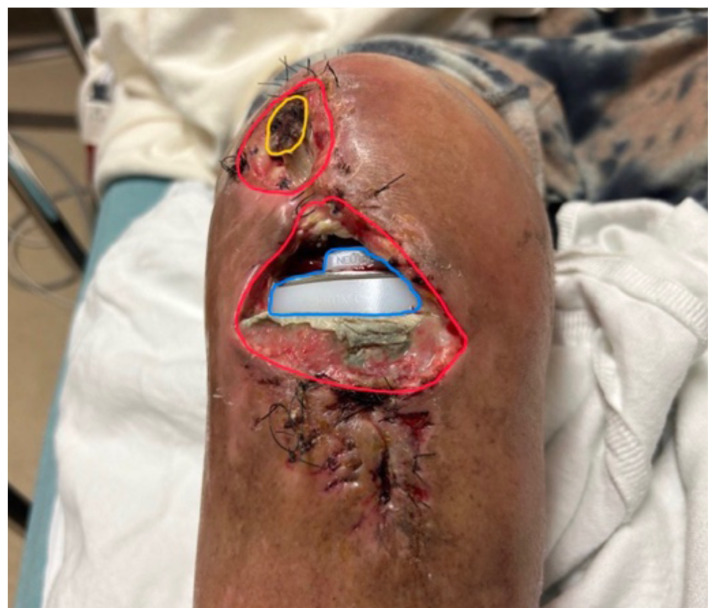


**Figure:**
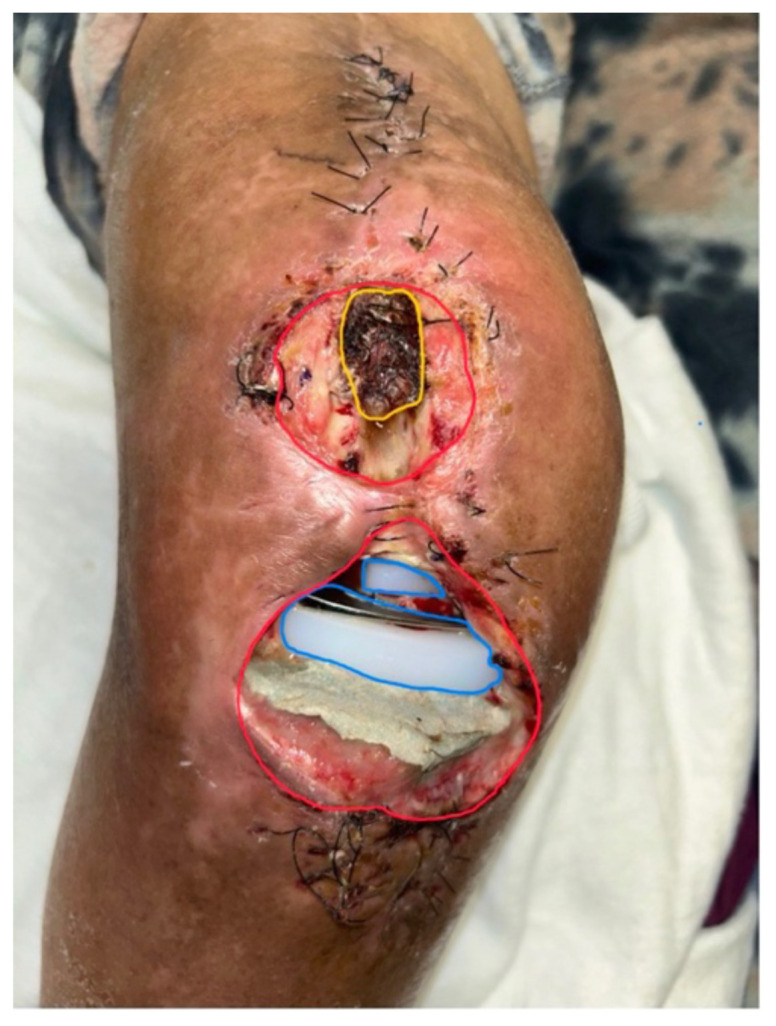


**Figure:**
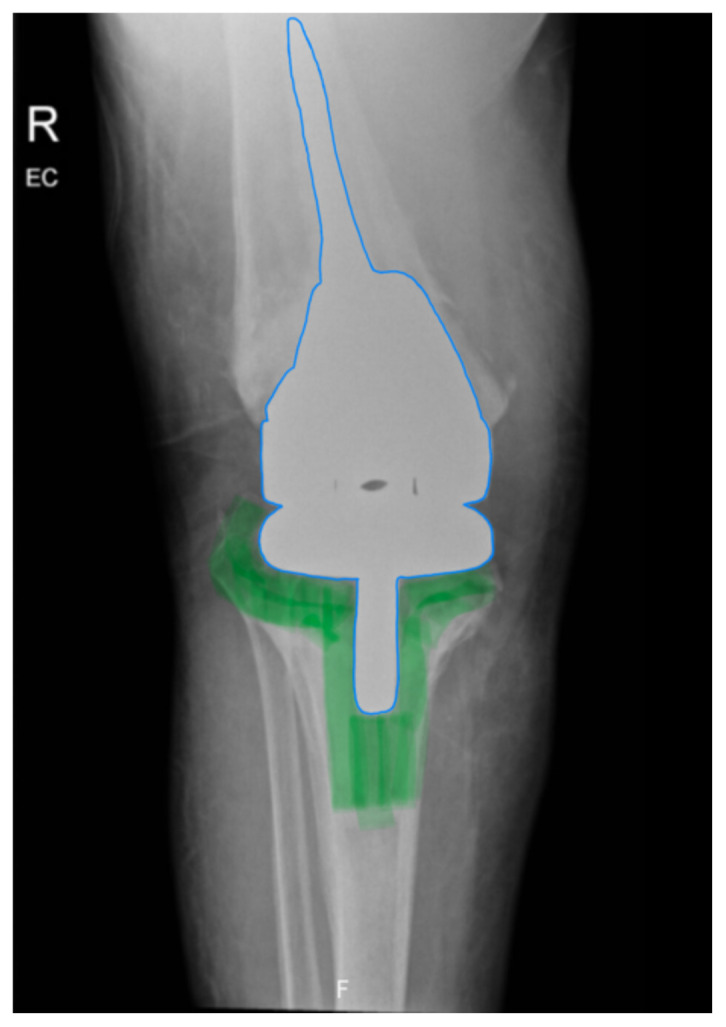


**Figure:**
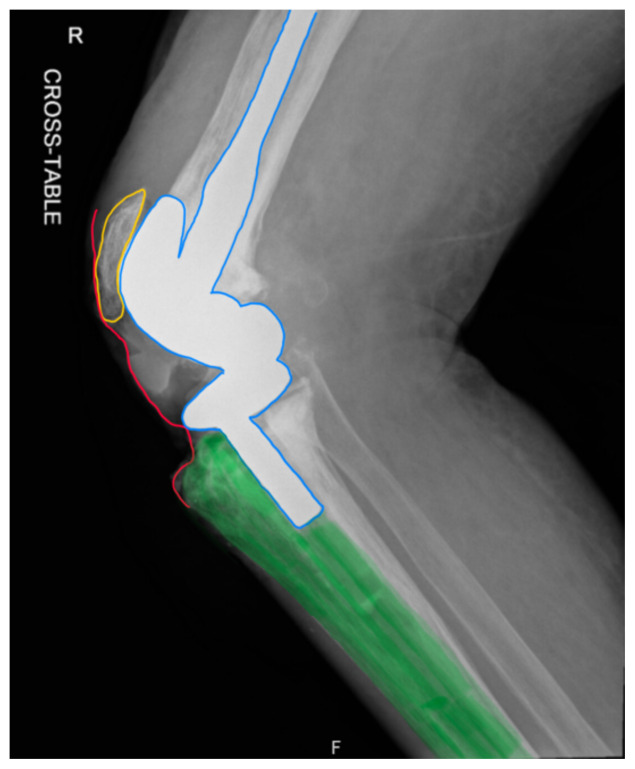


**Figure:**
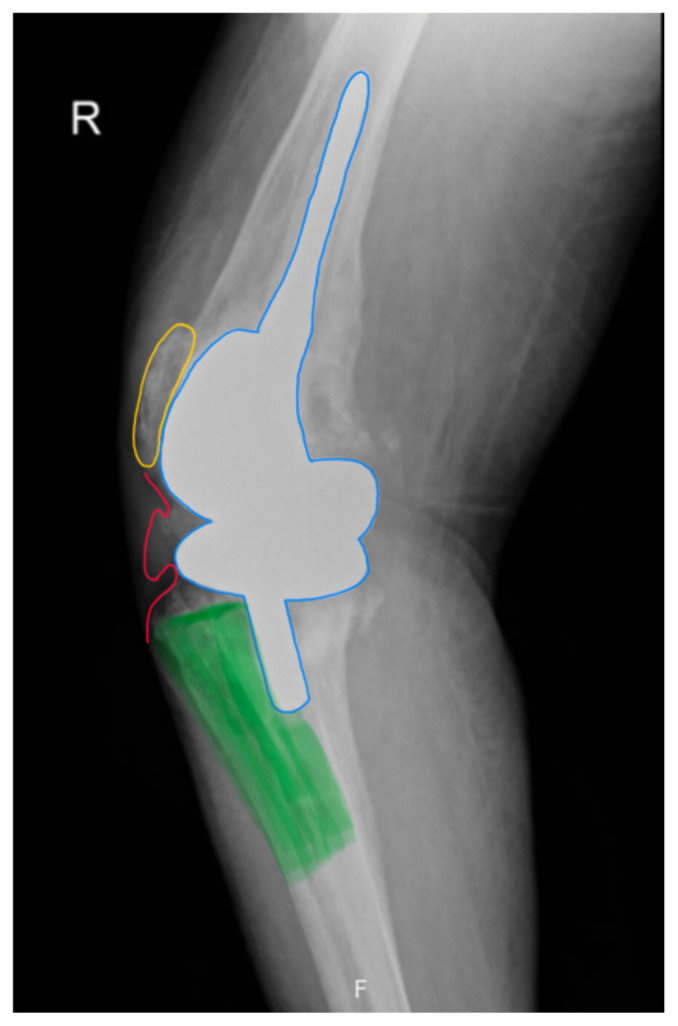


## Brief introduction

Annually, an estimated 750,000 total knee arthroplasties (TKA’s) are performed in the U.S. with a projected 1,194,000 to be performed in 2035.^1^ It is relatively common to see TKA patients in the emergency department, with between one in seven to one in ten patients presenting within 30 days of surgery.^2^ Presenting symptoms include pain (4.6–35%), “medical concerns” (5.6–24.5%), and swelling (1.4–17.5%).^3^ Despite their frequent presentation, true complications of these TKA patients in the Emergency Department are rare and include infection (0.5–1.9%) and acute dehiscence (0.05–0.1%).^4^–^5^ Often they present with erythema and with tension on the sutures. Of those infected TKAs, 0.025%–5.1% require amputation.^4^ As such, acute dehiscence, infection, and subsequent amputations are relatively rare yet devastating events following a TKA.

Case reports have been written about these rare TKA complications; however, no literature to date has been found that describes all three in the same patient. This case report describes an acute traumatic wound dehiscence in a TKA patient who presented to the emergency department as a chronically exposed and infected wound with prosthetic and skeletal exposure necessitating an amputation. Although this is an extreme and interesting case, it provides an opportunity for a discussion of the management of prosthetic joint infections (PJIs) versus native joint infections in the emergency department.

As the front door for medical care in the community, the emergency department evaluates many vulnerable populations. Often the progression of patient’s health and disease processes is heavily influenced by socioeconomic barriers discovered in the emergency department. These barriers include housing insecurity, disability, and substance use among many others. Of these, an estimated 5% of patients in the emergency department screen positive for housing insecurity. 5 Among all patients’ visits in the emergency department, 40% are people with disabilities and 4% have a substance abuse diagnosis in their treatment list.^6^,^7^ Often these barriers intersect and amplify each other to negatively impact patient care. This patient’s socioeconomic factors included housing insecurity, disability, and substance use which presented a clear challenge to her long-term care and follow-up of her TKA that is worth discussing how clinicians in the emergency department can intervene.

## Presenting concerns and clinical findings

A 63-year-old female with arthritis, polysubstance use, and housing insecurity presented with right knee pain. The pain started after a fall at home shortly after a replacement TKA three months ago. The patient was admitted twice at an outside hospital for wound dehiscence secondary to the original fall with multiple failed attempts at limb salvage therapy, leaving her with a chronically open wound. During both hospitalizations, the patient left against medical advice when scheduled for an above knee amputation (AKA).

The patient presented to our emergency department 11 weeks after the initial fall. She presented after a neighbor provided a “wellness” check to the patient earlier in the day and discovered a gaping wound over the patient’s knee. On initial evaluation, the patient was chronically ill-appearing in a wheelchair with saturated bandages covering her right knee and a chronically dehisced wound (red) with necrotic tissue and surrounding erythema. There was obvious exposure of the prosthetic (blue) with tibial and femoral involvement and a superiorly displaced palpable patella (yellow). She denied systemic infectious symptoms and was unable to ambulate or extend her knee.

## Significant findings

She presented afebrile (36.9°C) with a heart rate of 108 beats per minute, respiratory rate of 16 breaths per minute, and blood pressure 146/82. Her white blood cells (WBC) count was within range (5,200 mm^3^) and remaining blood counts and complete metabolic panel were unremarkable. The patient’s creactive protein (CRP) was 6.2mg/L (elevated), erythrocyte sedimentation rate (ESR) 29mm/hr (within normal limits) and lactate 2.3mEq/L (mildly elevated). Blood cultures showed no growth at 48 hours. Radiographs demonstrated intact prosthetic components (blue) without acute fracture, tibial peri-stem lucency (green) concerning for loosening or infection, as well as joint effusion, and soft-tissue swelling.

## Patient course

While in the emergency department, the patient had a leading differential of prosthetic joint infection. Although she presented tachycardic, she was otherwise negative for systemic inflammatory response syndrome (SIRS).^8^ She was started on intravenous (IV) piperacillin-tazobactam for the joint infection and overlying cellulitis. In the ED, she vocalized her wishes for nonoperative management citing she was scared to lose her leg and what her life would look like afterwards. She expressed that she kept leaving or declining amputation because she was her sole caretaker and had unstable housing. After a careful and emotional discussion on the severity of her condition, she elected for operative management. Orthopedics accepted the patient for definite management with an AKA. At the time of admission, the patient was understanding of her condition and expressed willingness to proceed with amputation. Unfortunately, she ultimately refused an AKA while an inpatient the day of the procedure and again a few days later after being rescheduled. This was consistent with a previous admission where she declined operative management. Her inpatient notes documented she had capacity during this visit to decline. She was seen by social work and case management to aid with substance abuse resources, housing insecurity, and discharge planning. Unfortunately, she was denied Skilled Nursing Facility due to her polysubstance abuse. The admitting teams discharged the patient after she had completed two weeks of IV antibiotics. Upon discharge to home, the patient was given an outpatient orthopedic referral and wound care instructions with supplies for daily saline flushes and kerlix wraps. It is unclear if home health was able to be arranged or how she would receive transportation to her appointment.

## Discussion

This case report highlights an extreme and unique instance of an acute traumatic dehiscence following a TKA that resulted in chronic wound dehiscence and PJI. While this is a very extreme case not commonly seen in ERs, it presents an opportunity to discuss native vs prosthetic joint infections (PJI), social factors to consider in caring for vulnerable patients, and a discussion on capacity.

Septic arthritis of a native joint presents as an acute onset, a “hot” swollen joint with pain with motion and often an inability to bear weight on the affected extremity.^9^ The general presenting findings and their sensitivities are listed in Table 1; however, none are sufficient to diagnose septic arthritis.^10^ Laboratory work including serum markers and the gold standard synovial fluid analysis are more often necessary, as listed in Table 2.^10^ From the table, many of the serum markers are non-specific, i.e., erythrocyte sedimentation rate (ESR) can be elevated in many conditions (including infection or gout), and the specificities for ruling out septic arthritis in both presenting findings and serum markers are poor. A procalcitonin has shown to be a promising serum marker with a relatively high sensitivity and high specificity in the proper clinical context but can still be non-specific.^11^ A clinician should use their judgment when interpreting these labs in the right clinical context to make a diagnosis since there is not clear diagnostic criteria.^10^ Currently, synovial fluid WBC, polymorphonuclear neutrophils (PMN), and cultures are considered of most value.^10^

One important consideration in diagnosing septic arthritis is ruling out crystal-induced arthritis. This should be done via obtaining synovial fluid analysis with gram stain, crystals, cell count, and culture. Differentiation should also be made between gonococcal and nongonococcal septic arthritis which can be differentiated by gram stain; however, antibiotic therapy should not be withheld if there is suspicion for gonococcal exposure.^12^

In contrast, PJIs present with a more indolent course with progressively worsening pain and nonspecific signs of erythema, a low-grade fever, and joint stiffness.^13^ The variable presentation and more insidious presentation of PJI may lie in the slower biofilm formation over its prosthetic material before tissue propagation.^13^,^14^ This compares to quicker bacterial invasion and destruction of the synovium and cartilage in native septic joints.^15^ As such, septic arthritis in a native joint is considered a more acute medical emergency due to the impending tissue and structure compromise not present in PJIs.^13^

**Image 1:**
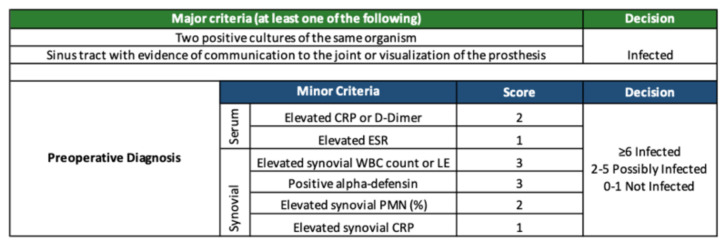
An updated evidence-based scoring tool from MSIS for PJIs.^17^

Specific diagnostic criteria for PJIs have been written, although have varied over the years.^16^ A 2018 study outlines a scoring tool (see Image 1) gaining favor with a sensitivity and specificity of 97.7% and 99.5%, respectively.^17^ This can be seen as an upgraded version of the commonly used Musculoskeletal Infection Society (MSIS) criteria for periprosthetic joint infection (PJI) in 2011. Under this tool, this patient’s pre-operative status met one major criterion (sinus tract with joint visualization) and a minor criteria score of two (elevated CRP), without synovial fluid analysis. Although this patient’s presentation was extreme, this scoring tool provides a standardized evidence-based approach to diagnosing prosthetic joint infections of the knee and a contrast to that for septic arthritis.^13^

The simplified goals of managing both types of joint infections are to restore joint function, decrease pain, and eradicate the infection. For native joint septic arthritis, this involves emergent recognition, empiric antibiotics, and joint drainage to avoid impending surrounding native structure damage.^18^ Antibiotic selection is differentiated between gonococcal vs nongonococcal septic arthritis as noted in Table 3. Differentiating these can be logistically difficult in the emergency department, and thus administering both vancomycin and ceftriaxone for broad empiric coverage can be started.^12^ Careful consideration should be made for other antibiotic regimens for patient risk factors including pre-existing medical conditions, prior antibiotic use, age, and likely pathogen. Joint drainage is necessary because the infection is like a closed abscess. For easy access joints (knee, elbow), this can be done through aspiration in consultation with orthopedics following the saying “drain to dryness.” 19

In comparison, PJI patients have a less urgent management timeline due to less threat of those native structures.^20^ Management is more individualized but usually includes initial empiric antibiotics (after blood or synovial samples) and debridement by an orthopedist. Empiric antibiotics include vancomycin and piperacillin-tazobactam.^21^ If warranted, operative management is tiered, with a combination of debridement, exchange surgery, or removal and reimplantation based on the orthopedic surgeon’s preference, patient candidacy, and available surrounding tissue.^22^ The main difference here is that native septic arthritis should be considered a limb-threatening emergency while PJI should under most circumstances only need to be managed urgently.^23^

It is only in extreme cases and as a last resort that amputation is actually necessary for PJI.^24^ Scenarios for amputation include persistent or uncontrollable infection, massive tissue destruction, arterial occlusion, bone loss, and periprosthetic fracture when other means have failed.^25^ As noted earlier, a 2021 systematic review found that of all complicated infected TKA’s, 5.1% resulted in amputation.^26^ For this patient, uncontrollable infection, massive tissue destruction, and previous operative failures necessitated this extremely rare step. She was repeatedly offered amputation by multiple surgeons as well as offered resources and support by social work and case management but ultimately declined surgery.

Overall, the mortality rates in PJI range from 2.7–18% while native septic arthritis ranges from 11–30%.^13^ Furthermore, the risk of permanent functional loss of the joint is nearly 40% for native septic arthritis and can even result in amputation. Although this extreme case points to the rare times PJI turns severe, this overall comparison shows that native septic arthritis should be handled more emergently in most cases for its high mortality and risk of joint function.

Turning the discussion to social factors that impact vulnerable patients, this patient had several socioeconomic barriers that played into the medical course. First, she had a history of substance abuse. It is not clear (due to the patient receiving care at multiple facilities) but seems that at one point she was not using any substances but relapsed after the initial TKA. In addition to polysubstance abuse, she also had no support system to lean on. It was only because of a neighbor checking in on her that she presented to the emergency department for this visit. Then there is the matter of her being wheelchair bound. This severely limited her ability to follow up and find transportation. Finally, she also struggled with housing insecurity. Although living in an apartment at the time of evaluation, she did make note of moving frequently. This played a part with her initial wound care months ago, having moved and not updating her address to continue receiving care. The patient believed her substance use, lack of support, disability, and risk of losing housing would only be worsened with an amputation. Thus, despite this being her only medical option, she kept declining.

Having an amputation would severely complicate this picture for her. This serves as a stark reminder for physicians that medical decisions need to include the whole patient, not just the medical problem at hand. This also points to a broader medical system that has large deficits in supporting vulnerable patients with multiple interesting social determinants. One way the emergency department can help mitigate and help vulnerable patients is early social work and case management involvement. Particularly, when giving handoff to the inpatient team, an emphasis on the severe barriers helps orient the long-term care goals for the patient and inpatient team. This would also include sharing the discussions as initiated in the department about operative intervention. As the safety net for vulnerable patients in the community, it is the duty of emergency physicians to advocate and initiate multidisciplinary care.

Finally, assessment of patient capacity played an important role in this case while the patient stayed in the hospital deciding on her ultimate treatment plan. There are four components of capacity: understanding of the condition, appreciation of any consequences, reasoning of the decision, and expression of a clear choice. If a patient is deemed to have capacity from these criteria, it is the duty of the physician to proceed with their decision. This patient was able to state she had an infection of her knee and that in not treating the infection via amputation, the infection would likely spread. Her reasoning to decline operation was clear, consistent, and based on her being scared with the socioeconomic factors discussed above. It should be noted though that just because she had capacity to decline some aspects of her treatment course, she should not be considered ineligible for other care options. Namely, she continued to receive a two-week course of IV antibiotics while inpatient.

## Conclusion

Our patient’s unique presentation of a dehisced and infected TKA in the emergency department provides a unique opportunity to compare PJIs versus native joint infections. Native joint septic arthritis is more emergent in nature while in the emergency department due to the threat of surrounding structures to native joints and higher mortality compared to PJI. However, the individuality of each patient, the timeline of infection, and other factors must be considered closely with orthopedic colleagues.

In addition, clinicians must work alongside the patient in assisting them in all aspects of their health including socioeconomic and must be empathetic to patients with decision-making capacity who make decisions outside of medical advice. This vulnerable patient had several barriers that prevented long-term and consistent support. Early intervention of social work, case management, and clear handoff to the inpatient team of these barriers is of extreme value in the long-term.

## Supplementary Information

























## Figures and Tables

**Table 1:** Common presenting findings in native joint septic arthritis and their sensitivity.^10^

Presenting Finding:	Sensitivity %
Fever	44.0–97.0
New Joint Swelling	77.0
Joint Pain with Motion	100.0
Limited Joint Motion	92.0
Tender Joint	68.0–100
Joint Swelling	45.0–92.0

**Table 2:** Serum markers and synovial fluid analysis sensitivities and specificities for diagnosing septic arthritis of a native joint.^10^

Serum Marker:	Sensitivity %	Specificity %
WBC >10 × 109/L	60.0–90.0	36.0
ESR 15mm/hr	66.0	48.0
ESR >30mm/hr	76.0–97.0	29.0
CRP >10mg/L	87.0–91.0	15.0–39.0
Procalcitonin >0.3 ng/mL	73.0	94.0
Blood Culture	23.0–36.0	NA
**Synovial Fluid Analysis:**		
WBC >1.7×10^9^	94.0	88.0
PMNs >90%	59.0–70.0	60.0–83.0
Lactate >5.6mmol/L	67.0	72.0
Lactate Dehydrogenase (LDH) >250 U/L	100.0	51.0
Synovial Culture	75.0–95.0	NA

**Table 3:** Empiric antibiotics for native and prosthetic joint infections (From *Horowitz et. al* and *Le Vavasseur et. al*)

Native Joint Septic Arthritis	Empiric Antibiotics
Empiric	Vancomycin + Ceftriaxone
Gram Positive (Nongonococcal)	Vancomycin or rifampin
Gram Negative Cocci (Gonococcal)	Ceftriaxone
Gram Negative Rods (Pseudomonal)	Piperacillin-tazobactam
**PJI** Empiric	Vancomycin + piperacillin-tazobactam

## References

[1] Shichman I, Roof M, Askew N (2023). Projections and epidemiology of primary hip and knee arthroplasty in Medicare patients to 2040–2060. JBJS Open Access.

[2] Ravi B, Leroux T, Austin PC, Paterson JM, Aktar S, Redelmeier DA (2020). Factors associated with emergency department presentation after total joint arthroplasty: A population-based retrospective cohort study. Can Med Assoc Open Access J.

[3] Maldonado-Rodriguez N, Ekhtiari S, Khan MM (2020). Emergency Department presentation after total hip and knee arthroplasty: A systematic review. J Arthroplasty.

[4] Savvidis I, Rigkos D, Solovos E, Georgiannos D, Bisbinas I Above-knee amputation after total knee replacement infection: The unfortunate end of an Odyssey. Cureus.

[5] Ball MA, Sack DE, Druffner SA (2024). Characteristics and health care utilization of patients with housing insecurity in the ED. JAMA Netw Open.

[6] Peterson C, Li M, Xu L, Mikosz CA, Luo F (2021). Assessment of annual cost of substance use disorder in US hospitals. JAMA Netw Open.

[7] Rasch EK, Gulley SP, Chan L (2013). Use of emergency departments among working age adults with disabilities: a problem of access and service needs. Health Serv Res.

[8] Bone RC, Balk RA, Cerra FB (1992). Definitions for sepsis and organ failure and guidelines for the use of innovative therapies in sepsis. American College of Chest Physicians/Society of Critical Care Medicine Consensus Conference. Crit Care Med.

[9] Hassan AS, Rao A, Manadan AM, Block JA (2017). Peripheral bacterial septic arthritis: Review of diagnosis and management. JCR J Clin Rheumatol.

[10] Carpenter CR, Schuur JD, Everett WW, Pines JM (2011). Evidence-based diagnostics: Adult septic arthritis. Acad Emerg Med Off J Soc Acad Emerg Med.

[11] Maharajan K, Patro DK, Menon J (2013). Serum Procalcitonin is a sensitive and specific marker in the diagnosis of septic arthritis and acute osteomyelitis. J Orthop Surg.

[12] Horowitz DL, Katzap E, Horowitz S, Barilla-LaBarca ML (2011). Approach to septic arthritis. Am Fam Physician.

[13] Roerdink RL, Huijbregts HJ, van Lieshout AW, Dietvorst M, van der Zwaard BC (2019). The difference between native septic arthritis and prosthetic joint infections: A review of literature. J Orthop Surg.

[14] Tande AJ, Patel R (2014). Prosthetic joint infection. Clin Microbiol Rev.

[15] García-Arias M, Balsa A, Mola EM (2011). Septic arthritis. Best Pract Res Clin Rheumatol.

[16] Kim SJ, Cho YJ (2021). Current guideline for diagnosis of periprosthetic joint infection: A review article. Hip Pelvis.

[17] Parvizi J, Tan TL, Goswami K (2018). The 2018 definition of periprosthetic hip and knee infection: An evidence-based and validated criteria. J Arthroplasty.

[18] Coakley G, Mathews C, Field M (2006). BSR & BHPR, BOA, RCGP and BSAC guidelines for management of the hot swollen joint in adults. Rheumatology.

[19] Earwood JS, Walker TR, Sue GJ (2021). Septic arthritis: Diagnosis and treatment. Am Fam Physician.

[20] Long B, Koyfman A, Gottlieb M (2019). Evaluation and management of septic arthritis and its mimics in the Emergency Department. West J Emerg Med.

[21] Le Vavasseur B, Zeller V (2022). Antibiotic therapy for prosthetic joint infections: An overview. Antibiotics.

[22] Nair R, Schweizer ML, Singh N (2017). Septic arthritis and prosthetic joint infections in older adults. Infect Dis Clin North Am.

[23] Luthringer TA, Fillingham YA, Okroj K, Ward EJ, Valle CD (2016). Periprosthetic joint infection after hip and knee arthroplasty: A review for emergency care providers. Ann Emerg Med.

[24] Osmon DR, Berbari EF, Berendt AR (2013). Diagnosis and management of prosthetic joint infection: Clinical practice guidelines by the Infectious Diseases Society of Americaa. Clin Infect Dis.

[25] Sierra RJ, Trousdale RT, Pagnano MW (2003). Above-the-knee amputation after a total knee replacement: Prevalence, etiology, and functional outcome. J Bone Joint Surg Am.

[26] Mousavian A, Sabzevari S, Ghiasi S (2021). Amputation as a complication after total knee replacement, is it a real concern to be discussed?: A systematic review. Arch Bone Jt Surg.

